# The Perceived Match Between Observed and Own Bodies, but Not Its Accuracy, Is Influenced by Movement Dynamics and Clothing Cues

**DOI:** 10.3389/fnhum.2021.701872

**Published:** 2021-07-28

**Authors:** Lize De Coster, Pablo Sánchez-Herrero, Jorge López-Moreno, Ana Tajadura-Jiménez

**Affiliations:** ^1^DEI Interactive Systems Group, Department of Computer Science and Engineering, Universidad Carlos III de Madrid, Madrid, Spain; ^2^Seddi Labs, Madrid, Spain; ^3^Multimodal Simulation Lab, Department of Computer Science and Architecture, Computer Systems and Languages, Statistics and Operative Investigation, Universidad Rey Juan Carlos, Madrid, Spain

**Keywords:** body representation, body perception, bodily self-awareness, movement, self-esteem, avatar

## Abstract

Own-perceived body matching – the ability to match one’s own body with an observed body – is a difficult task for both general and clinical populations. Thus far, however, own-perceived body matching has been investigated in situations that are incongruent with how we are used to experience and perceive our body in daily life. In the current study, we aimed to examine own-perceived body matching in a context that more closely resembles real life. More specifically, we investigated the effects of body movement dynamics and clothing cues on own-perceived body matching. We asked participants to match their own body with an externally perceived body that was a 3D-generated avatar based on participants’ real bodies, fitted with a computer-generated dress. This perceived body was (1) either static (non-walking avatar) or dynamic (walking avatar), (2) either bigger, smaller, or the same size as participants’ own body size, and (3) fitted with a dress with a size either bigger, smaller, or the same as participants’ own dress size. Our results suggest that movement dynamics cues did not improve the accuracy of own-perceived body matching, but that confidence about dress fit was higher for dynamic avatars, and that the difference between dynamic and static avatars was dependent on participants’ self-esteem. Furthermore, when participants were asked to rate the observed body in reference to how they wanted to represent themselves to others, dynamic avatars were rated lower than static avatars for the biggest-sized bodies only, possibly reflecting the influence of movement cues on amplifying socio-cultural stereotypes. Finally, while smaller body/dress sizes were systematically rated higher than bigger body/dress sizes for several self-report items, the interplay between body and dress size played an important role in participants’ self-report as well. Thus, while our research suggests that movement and garment dynamics, allowing for realistic, concrete situations that are reminiscent of daily life, influence own-body perception, these cues did not lead to an improvement in accuracy. These findings provide important insights for research exploring (own-) body perception and bodily self-awareness, with practical (e.g., development of online avatars) and clinical (e.g., anorexia nervosa and body dysmorphic disorder) implications.

## Introduction

We experience and interact with the world through our body. In order to do so efficaciously and efficiently, humans need to be able to accurately and dynamically perceive their own body. Own-body perception has been extensively investigated using body illusions where the perception of one’s body deviates from the physical one (for a review see Kilteni et al., 2015). These include body distortion illusions, in which the size or posture of the body or its body parts are perceived as distorted (e.g., Goodwin et al., 1972; Ramachandran and Hirstein, 1998); out-of-body illusions, in which people perceive their self to be dislocated from their own body and/or people look at their body from a distance (e.g., Ehrsson, 2007; Lenggenhager et al., 2007); and body ownership illusions, in which non-bodily objects are perceived as a part of one’s own body (e.g., Botvinick and Cohen, 1998; Petkova and Ehrsson, 2008; Dummer et al., 2009; Peck et al., 2013; Maselli and Slater, 2014). These illusions demonstrate that the sense of body ownership, defined as the experience of one’s body and its body parts as one’s own, and necessary to move through the world and interact with others (Martin, 1995; Gallagher, 2000; Ehrsson, 2012; Gallagher and Daly, 2018), is a dynamic and malleable process that is determined by multisensory integration mechanisms (Ehrsson, 2012; Kilteni et al., 2015; Ehrsson and Chancel, 2019; Chancel and Ehrsson, 2020).

In addition to perceiving our own body from within through the integration of multisensory and sensorimotor inputs (Kilteni et al., 2015), own-body perception also takes places when confronted with the task of matching an externally perceived body with our own. This matching of our own body with an externally perceived body (own-perceived body matching) has been shown to be largely inaccurate, with people systematically over-estimating (Hashimoto and Iriki, 2013; Linkenauger et al., 2017; Sadibolova et al., 2019) or under-estimating (Valentina Tovée et al., 2003; Cazzato et al., 2016b; Ralph-Nearman et al., 2019) their body shape and size. These distortions in our body image have been measured both explicitly (Hashimoto and Iriki, 2013; Linkenauger et al., 2017; Pitron and de Vignemont, 2017; Sadibolova et al., 2019) and implicitly (Longo and Haggard, 2010, 2011; Maister et al., 2021). Importantly, they impact general well-being and have been linked to various clinical disorders (Stice and Shaw, 2002; Kaplan et al., 2013; Dakanalis et al., 2016). Furthermore, this inability to match own and perceived body has several practical implications, such as for the design of self-avatars for online gaming (Ducheneaut et al., 2009) and retail (Merle et al., 2012) experiences. The latter, for example, suffers from general dissatisfaction with purchased items and high return rates (Gallup, 1970; Petersen and Kumar, 2009; Saarijärvi et al., 2017), which have been partly attributed to a lack of resemblance between consumers and their online model/avatar (Kim and Forsythe, 2008). Nevertheless, despite its clinical and practical importance, this form of own-body perception, which involves matching an externally perceived body with one’s own, has remained difficult to improve.

In a study comparing healthy controls with individuals diagnosed with anorexia nervosa, researchers achieved this seemingly difficult task by generating personalized realistic avatars using a combination of 3D scanning and computer-generated imagery (CGI) techniques (Cornelissen et al., 2017). While an over-estimation of own body measurements was still observed in the group of individuals diagnosed with anorexia nervosa, the healthy control group showed accurate body size estimation. The authors suggested that their combined 3D-CGI method might be less prone to visual artifacts and may provide a clearer insight into the size and shape that someone considers him/herself to be. Additionally, they argue that contextualizing own-body evaluation in ecologically valid situations (e.g., looking in the mirror) is vital for future research in the field. While they suggest that the only way to truly achieve this is by allowing participants to inhabit a personalized 3D avatar in whom participants can manipulate body changes in real time, this method has rendered conflicting results (Piryankova et al., 2014; Preston and Ehrsson, 2014; Dakanalis et al., 2017) and poses practical challenges that are difficult to implement in daily life (e.g., the widespread availability of at-home technology to inhabit 3D avatars). Furthermore, during body perception/estimation experiments, own-perceived body matching is often performed in a way that is incongruent with how we are used to experiencing and observing our own body in daily life. First, while we are used to experience our own body in movement, movement dynamics have thus far not been included when investigating own-perceived body matching, although action and motor experience have been shown to be important in the development and maintenance of body ownership (e.g., Dummer et al., 2009; Nava et al., 2018). Second, the avatar/model bodies during own-perceived body matching are usually presented either without clothing (e.g., De Coster et al., 2020) or with static clothing that does not provide additional cues (e.g., wrapping of different sizes of clothing around the body, movement of clothing when body moves) for body size estimation (e.g., Cornelissen et al., 2017; Mölbert et al., 2018; Thaler et al., 2018; Sadibolova et al., 2019). While it has been shown that dynamics play an important role in the perception of clothing (Aliaga et al., 2015) and that observers are able to infer certain body properties (e.g., body stiffness) from clothing dynamics (Romero et al., 2020), as well as the clothing’s mechanical properties (Bi and Xiao, 2016), the question whether body size can be predicted by these dynamics and whether own-perceived body matching would be improved by these additional cues remain open questions. In sum, while movement and clothing dynamics likely play an important role in own-body perception in daily life, they have thus far not been investigated.

In the current study, we built upon the idea of emulating real-life practical situations when investigating own-body perception in the context of matching own with a perceived body. More specifically, the aim of this research was to systematically examine the influence of movement dynamics and clothing, two factors that are usually present when we perceive our own body in daily life, on own-perceived body matching. While it has been claimed that we do not have access to observing our body in motion (Kadambi and Lu, 2018), we argue that we rarely observe our own bodies and the accessories that come along with it in purely static positions (e.g., twisting and turning in front of a mirror). Furthermore, while the recognition of our body in motion depends on the integration of the combination of visual, somatosensory, proprioceptive, and motor information (Myers and Sowden, 2008), as well as auditory information (Tajadura-Jiménez et al., 2015), we believe that the contribution of visual motion cues alone may still be of relevance to this recognition process. In order to achieve this aim, we created several realistic 3D avatars of different sizes based on participants’ bodies using Skinned Multi-Person Linear modeling (SMPL; Loper et al., 2015). This parametric modeling method is thought to be more accurate and easier to use than other methods, partly because it avoids the intense manual effort inherent to commercial approaches (e.g., CGI). In accordance with a previous study using a similar method (De Coster et al., 2020) and previous research using other techniques (Valentina Monteath and McCabe, 1997; Tovée et al., 2003; Johnson et al., 2008; Fikkan and Rothblum, 2012; Cazzato et al., 2015; Spahlholz et al., 2016; Robinson and Kersbergen, 2017; Steinsbekk et al., 2017; Ralph-Nearman et al., 2019), we expected participants to not be able to accurately match their own with a perceived body. More specifically, we expected them to show a preference for smaller- compared to bigger-sized avatars, irrespective of their own body size. Importantly, we contextualized the task of matching own and perceived body in a real-life situation by (1) comparing the accuracy of matching participants’ own with a perceived avatar’s body that was either static or dynamic (walking avatar), (2) fitting the observed model/avatar with a computer-simulated dress in different sizes, and (3) specifically asking participants about their wish to use the perceived model/avatar for online shopping (De Coster et al., 2020). Concerning the effect of movement dynamics, we hypothesized that the addition of dynamic movement cues would increase participants’ ability to accurately determine their own body size/shape given the additional information that these movement cues provide and the resemblance to our everyday real-life environment. This comparison of static vs. dynamic avatars was our main effect of interest, since we expected these findings to render important insights into the role of action cues in own-body perception and bodily self-awareness, with both clinical and practical implications. To further examine these implications, we investigated whether this effect of movement dynamics was modulated by bodily self-esteem and personality differences given that previous research has shown that both self-esteem (e.g., Maister et al., 2021) and personality variables (e.g., De Coster et al., 2020) influence body size estimation. Both healthy (Cornelissen et al., 2013) and clinical (Gardner and Brown, 2014) populations with negative attitudes toward their own body weight, as well as healthy populations scoring higher on neuroticism (Hartmann and Siegrist, 2015), have been shown to overestimate their own body size. We consequently expected that the addition of dynamic cues – which we hypothesized would lead to more accurate body size estimation – might have a different effect (e.g., due to differences in the processing of bodily information; Irvine et al., 2019) for participants with certain personality traits (e.g., neuroticism) and participants scoring low on bodily self-esteem measures, compared to other participants. Finally, we added a dress simulation in different sizes to ensure that the perception of the avatar’s body was congruent with how we generally observe our bodies in everyday situations where we mostly perceive ourselves with, rather than without, clothes (note that this dress simulation was also influenced by the body’s movement dynamics). Thus, we expected that a correct dress size would improve the detection of participants’ own body size.

## Materials and Methods

### Participants

Sample size was dictated by a Bayesian approach using JASP (JASP Team, 2020). Participants were recruited from a subject pool of participants who participated in previous experiments, with the following inclusion criteria: (1) given that the experimental stimuli had to be based on videos of participants’ actual bodies (see below), participants were only eligible if such videos were available since the COVID-19 pandemic and the videos’ specific requirements (e.g., correct distance between the participant and the camera, no background items present, specific clothing for the participant to wear) made it impossible for us or for the participants themselves to record new videos, (2) in order to be able to model both a dress size below and above participants’ real dress size, only participants with a self-reported dress size of 38 or 40 (EU sizes) were eligible (only EU dress sizes 36, 38, 40, and 42 were available to be modeled), (3) to exclude gender effects (He et al., 2020), all participants had to be female. Considering these criteria, the size of our initial available subject pool was 20. We scheduled to test 15 participants, and planned to check the Bayes Factor (BF; prior based on a Cauchy distribution, default scale of 0.707, zero-centered) after data collection for this group was completed. If a stopping criterion had not been reached, we planned to repeat this procedure for the additional five participants, and expand the subject pool if necessary. The stopping criteria included: (1) the BF reached the threshold for moderate evidence to either support (BF_10_ < 1/3) or reject (BF_10_ > 3) the null hypothesis for the effect of dynamic vs. static avatars (our main effect of interest) for all self-report items, (2) the pre-specified end date (30/06/2020) had been reached. The experiment was terminated due to reaching the first criterion, and data collection was halted at 15 participants.

Fifteen adults (age in years: range = 18–28, *M* = 21.60, SD = 2.65; 11 participants with dress size 38, four participants with dress size 40), all female and residing in Spain, participated in the study in exchange for a gift card of 10 euros. Body mass index (BMI) in our sample ranged between 19.3 and 24.1 (*M* = 21.49, SD = 1.55), which lies within the healthy range (18.5–24.9) as defined by the World Health Organization. One participant scored more than two standard deviations below the sample average on all subscales of the bodily self-esteem questionnaire (see below; see Table 1 for questionnaire data). Removing this participant from the analyses did not change the results. The study was conducted in accordance with the 1964 Declaration of Helsinki and was granted ethical approval by the local ethics committee at Universidad Carlos III de Madrid. All participants provided informed written consent beforehand.

**TABLE 1:** Mean (*M*) and standard deviations (SD) for the subscales of the Body Esteem Scale for Adolescents and Adults (BESAA; rated on a scale from 0 to 4) and the Big 5 Inventory-10 (BFI-10; rated on a scale from 1 to 5).

Questionnaire subscale	*M*	SD
BESAA Appearance	2.54	0.76
BESAA Attribution	2.47	0.50
BESAA Weight	2.72	0.95
BFI-10 Extraversion	3.00	0.80
BFI-10 Agreeableness	2.90	0.64
BFI-10 Conscientiousness	3.77	0.77
BFI-10 Neuroticism	2.93	0.79
BFI-10 Openness	3.73	0.95

### Stimuli and Apparatus

**FIGURE 1:**
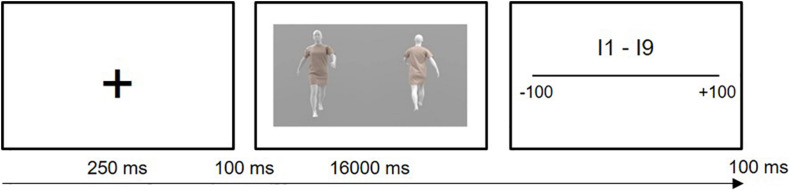
Schematic overview of the experimental procedure. During dynamic trials, a 4 s video of a walking avatar was shown in a loop four times for 16 s. During static trials, two frames were selected out of these 4 s videos, and were then also looped for 16 s. Participants were shown both the front and back view of the avatars in both types of trials.

Figure 1 shows an example frame of the experimental stimuli within the experimental procedure. Example videos (all Body/Dress size combinations are represented in one video for the dynamic and static condition separately) can be found in the Supplementary Material.

After obtaining a 360° full-body capture of participants, existing software COLMAP (Schönberger and Frahm, 2016; Schönberger et al., 2016) and custom-made scripts were used to create an avatar representing participants’ real bodies. This avatar was represented using SMPL (Loper et al., 2015) which includes several parameters to modify the avatar mesh. For each participant, different avatars were created by increasing or decreasing the second shape parameter, which primarily reflects changes in waist circumvention (although the avatar’s full body changed proportionally with respect to participants’ original body size, i.e., Body size 0). This resulted in three different avatars per participant: an avatar with a body size smaller than participants’ original body size (Body size −1; approximately 4 cm waist reduction), an avatar representing participants’ original body size (Body size 0), and an avatar with a body size bigger than participants’ original body size (Body size +1; approximately 4 cm waist increase; for full details on the avatar creation process see De Coster et al., 2020).

Additionally, a digital dress was created after extracting the patterns and creating 3D meshes from a real dress that was bought in different sizes (36, 38, 40, and 42). The patterns and initial resting position of the virtual dress were created with CLO3D^1^. Before extracting the dress’ 3D mesh, the dress was partially inflated to separate it from the skin of the avatar mesh, to ensure that there were no initial collisions in the simulation. Similar to the body size manipulation, different dress sizes were created: a dress size that was a size smaller than participants’ original dress size (Dress size −1), a dress size that reflected participants’ original dress size (Dress size 0), and a dress size that was a size bigger than participants’ original dress size (Dress size +1). This resulted in nine body/dress size combinations that were randomized per participant.

In order to allow for dynamic stimuli that represented real-life body/dress behavior during action movement, a walking animation was simulated for all avatars (Varol et al., 2017). The dress simulation was added using the simulation engine ARCSim, which allows for fine details and the preservation of fine-scale dynamic behavior (Narain et al., 2012, 2013; Pfaff et al., 2014). The application of one of the default materials (Wang et al., 2011) resulted in a sequence of meshes that represented the dress in different states of the avatar animation. Subsequently, videos of front and back views of the walking avatars with the dress simulation were rendered using Maya (Autodesk, 2019), and combined into one 4-s video in MATLAB (front view of the avatar on the left side, back view of the avatar on the right side).

Finally, two different video types (1,280 × 720 pixels) were created that were used as experimental stimuli. For the dynamic stimuli, videos of the walking avatars were looped four times to allow for sufficient time to inspect both the front and back view of the avatars (16 s; this duration was selected based on a pilot where several durations were tested). For the static stimuli, two frames (one frame where the avatar has the left foot in front, and another frame where the avatar has the right foot in front) were selected out of the original 4-s videos using Matlab. These frames were combined into a 4-s video in which each frame was shown for 2 s, and looped four times such that the total duration of these static stimuli was equal to that of the dynamic stimuli (16 s).

### Self-Report Measures

As described above, an experimental trial consisted of participants observing one of the stimuli for 16 s. At the end of each trial, participants were presented with nine self-report items that had to be rated on a continuous scale from −100 to +100. These items were adapted from previous research (Jin, 2010; Latoschik et al., 2017; De Coster et al., 2020), and measured participants’ own body perception in terms of perceived match between the observed avatar’s body and their own, as well as participants’ preferences toward the observed avatar across different dimensions (see Table 2 for a description of the items). The items were always presented in the same order: “Dress,” “Dress confidence,” “Measurements,” “Measurements confidence,” “Body,” “Myself,” “Others,” “Attractiveness,” and “Rebrowse.” Explicit certainty judgments (i.e., items related to confidence) for the “Dress” and “Measurements” items were added given that research has shown that the reliability of perception across different decisions might be related to subjective rather than objective accuracy (Fairhurst et al., 2018).

**TABLE 2:** Description of the self-report items, in the order that they were administered at the end of each trial.

Item	Question/statement
Dress	How likely do you think it is that this dress fits you?
Dress confidence	How certain are you?
Measurements	How likely do you think it is that this avatar’s measurements correspond to your own?
Measurements confidence	How certain are you?
Body	I feel as if the body of the avatar is my own body
Myself	The avatar reflects how I consider myself to be
Others	I consider the avatar to reflect how I want to present myself to others
Attractiveness	How attractive do you find the woman represented by this avatar?
Rebrowse	How likely do you think it is that you would choose this avatar as your avatar for online shopping?

### Body Esteem and Personality Questionnaires

#### Body Esteem Scale for Adolescents and Adults

The Body Esteem Scale for Adolescents and Adults (BESAA) is a 23-item questionnaire that measures people’s affective attitudes toward their own bodies (Mendelson et al., 2010). The questionnaire is comprised of three subscales that address general feelings about one’s appearance (Appearance), evaluations attributed to others about one’s body appearance (Attribution), and satisfaction with one’s body weight (Weight). The questionnaire items are rated on a Likert scale from 0 to 4, with higher scores reflecting more positive attitudes. Cronbach’s α in the current study was 0.89 (Appearance), 0.74 (Attribution), and 0.95 (Weight).

#### Big 5 Inventory-10

The Big 5 Inventory-10 (BFI-10) is a 10-item version of the Big 5 Personality Test (Benet-Martínez and John, 1998) that measures personality traits. Items are rated on a Likert scale from 1 to 5, and they correspond to five subscales: Extraversion (Cronbach’s α 0.65), Agreeableness (Cronbach’s α 0.71), Conscientiousness (Cronbach’s α 0.67), Neuroticism (Cronbach’s α 0.54), and Openness (Cronbach’s α 0.88; Benet-Martínez and John, 1998; Rammstedt and John, 2007).

### Procedure

Gorilla Experiment Builder (Anwyl-Irvine et al., 2020) was used to create and host the experiment online. Participants were instructed to complete the experiment individually and in one setting (verified afterward by the experimenter by checking completion dates/times). Participants were then told that they would observe avatars of different sizes (based on their own body) wearing a dress, and that they would have to answer several questions about the avatars they observed. The experiment consisted of 72 randomized trials (four times nine static and nine dynamic videos of a combination of three different body and dress sizes). On each trial, a fixation cross was presented for 250 ms, and after a 100 ms blank screen, the avatar video was shown for 16,000 ms. Immediately after the end of this video, participants responded to the nine self-report items at their own pace (see Figure 1). After completion of the self-report items and an inter-trial interval of 100 ms, the next trial started. At the end of the experiment, participants filled in the body esteem and personality questionnaires and were instructed to contact the experimenter to receive their monetary compensation. The experiment had a maximum total duration of 30 min.

### Design and Data Analysis

Normality checks were performed with Shapiro-Wilks tests (all *p*s > 0.237). A 2 × 3 × 3 repeated-measures design was used for each self-report item separately, with three within-subject factors: Animation (Static vs. Dynamic), Body size (Body −1 vs. Body 0 vs. Body +1), and Dress size (Dress −1 vs. Dress 0 vs. Dress +1). Follow-up paired samples *t*-tests and correlations between effects of interest and questionnaire data were corrected for multiple comparisons using false discovery rate (fdr) correction. Data were analyzed using a frequentist approach in R (R Core Team, 2020) as well as a Bayesian approach in JASP (JASP Team, 2020). The latter approach was used to test (1) whether there was moderate to strong evidence to reject the null hypotheses under a Bayesian framework in case of a significant effect and (2) whether potential null results could be considered support for the absence of any effects. For the Bayesian analysis, we obtained *BF*_10_ – representing the observation of the data under the alternative hypothesis compared to the null hypothesis (Wagenmakers et al., 2018) – for each main and interaction effect. We employed a threshold of moderate evidence to support (*BF*_10_ < 1/3) or reject (*BF*_10_ > 3) the null hypothesis.

## Results

### Main Effects

**FIGURE 2:**
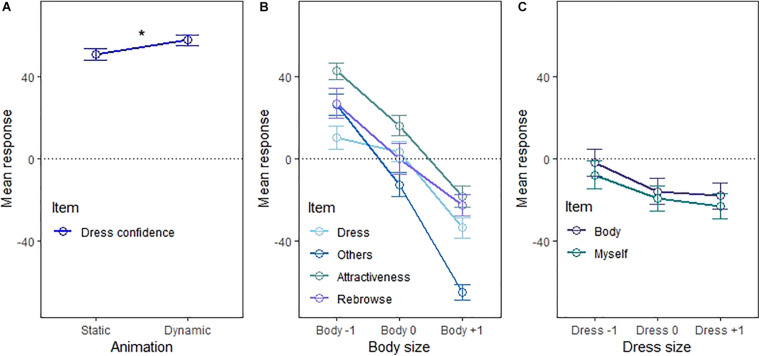
Main effects of **(A)** Animation, **(B)** Body size, and **(C)** Dress size. Dress = *How likely do you think it is that this dress fits you?*, Dress confidence = *How certain are you?*, Body = *I feel as if the body of the avatar is my own body*, Myself = *The avatar reflects how I consider myself to be*, Others = *I consider the avatar to reflect how I want to present myself to others*, Attractiveness = *How attractive do you find the woman represented by this avatar?*, Rebrowse = *How likely do you think it is that you would choose this avatar as your avatar for online shopping?* Body/Dress –1 = One body/dress size smaller than participants’ real body/dress size, Body/Dress 0 = Participants’ real body/dress size, Body/Dress +1 = One body/dress size bigger than participants’ real body/dress size.

The main effects of Animation, Body size, and Dress size are summarized in Figure 2. For Animation, a significant effect was observed for the “Dress confidence” item only [*F*(1,13) = 6.33, *p* = 0.026, $\eta_{p}^{2}$ = 0.33, *BF*_10_ = 3.137], indicating more confidence about dress fit for dynamic (*M* = 56.90, SD = 5.96) compared to static (*M* = 50.90, SD = 5.98) avatars (see Figure 2A). None of the other self-report items showed an effect of Animation (all *p*s > 0.253, all *BF*_10_ < 0.223).

For Body size, a significant effect was observed for the items “Dress” [*F*(2,12) = 5.86, *p* = 0.017, $\eta_{p}^{2}$ = 0.49, *BF*_10_ = 2.429e^+7^], “Others” [*F*(2,12) = 23.51, *p* < 0.001, $\eta_{p}^{2}$ = 0.80, *BF*_10_ = 3.090e^+28^], “Attractiveness” [*F*(2,12) = 17.87, *p* < 0.001, $\eta_{p}^{2}$ = 0.75, *BF*_10_ = 8.481e^+8^], and “Rebrowse” [*F*(2,12) = 4.09, *p* = 0.044, $\eta_{p}^{2}$ = 0.41, *BF*_10_ = 1.378e^+9^]. Figure 2B shows that a negative linear relationship was consistently observed for these items across the three body sizes.

Finally, for Dress size, a significant effect was found for the items “Body” [*F*(2,12) = 5.84, *p* = 0.017, $\eta_{p}^{2}$ = 0.49, *BF*_10_ = 0.530] and “Myself” [*F*(2,12) = 6.12, *p* = 0.015, $\eta_{p}^{2}$ = 0.50, *BF*_10_ = 0.511]. Similar to the effects of Body size, bigger dress sizes were rated lower than smaller dress sizes (see Figure 2C).

For significant pairwise comparisons that survived fdr-correction of the effects of Body and Dress size, see Table 3. Note that while the significant effects for Animation and especially Body size all reached the threshold of moderate evidence to reject the null hypothesis (set in the Bayesian analysis), this was not the case for the significant effects concerning Dress size.

**TABLE 3:** Pairwise comparisons of the main and interaction effects of Animation, Body size, and Dress size.

Effect	Item	Comparison	fdr-corrected *p*-value	Cohen’s *d*
Animation	Dress confidence	Static vs. Dynamic	0.026	1.01
Body size	Dress	Body −1 vs. Body +1	0.027	3.61
		Body 0 vs. Body +1	0.015	3.67
	Others	Body −1 vs. Body 0	0.001	3.97
		Body −1 vs. Body +1	<0.001	10.41
		Body 0 vs. Body +1	<0.001	5.85
	Attractiveness	Body −1 vs. Body 0	0.003	2.97
		Body −1 vs. Body +1	<0.001	6.96
		Body 0 vs. Body +1	<0.001	3.77
	Rebrowse	Body −1 vs. Body +1	0.021	5.02
		Body 0 vs. Body +1	0.021	3.84
Dress size	Body	Dress −1 vs. Dress +1	0.015	2.66
	Myself	Dress −1 vs. Dress +1	0.009	2.70
Animation x Body size	Others	Body +1: Static vs. Dynamic	0.033	0.22
Body size x Dress size	Dress	Dress −1: Body −1 vs. Body +1	0.014	1.49
		Dress −1: Body 0 vs. Body +1	0.014	1.08
		Dress 0: Body 0 vs. Body +1	0.015	0.74
	Dress confidence	Body +1: Dress −1 vs. Dress +1	0.030	0.58
	Measurements confidence	Body +1: Dress −1 vs. Dress +1	0.024	0.49
	Myself	Dress −1: Body −1 vs. Body +1	0.045	1.31
		Dress −1: Body 0 vs. Body +1	0.045	0.83

*Dress = How likely do you think it is that this dress fits you?, Dress confidence = How certain are you?, Measurements confidence = How certain are you? (In response to How likely do you think it is that this avatar’s measurements correspond to your own?), Body = I feel as if the body of the avatar is my own body, Myself = The avatar reflects how I consider myself to be, Others = I consider the avatar to reflect how I want to present myself to others, Attractiveness = How attractive do you find the woman represented by this avatar?, Rebrowse = How likely do you think it is that you would choose this avatar as your avatar for online shopping? Body/Dress −1 = One body/dress size smaller than participants’ real body/dress size, Body/Dress 0 = Participants’ real body/dress size, Body/Dress +1 = One body/dress size bigger than participants’ real body/dress size.*

### Interaction Effects

**FIGURE 3:**
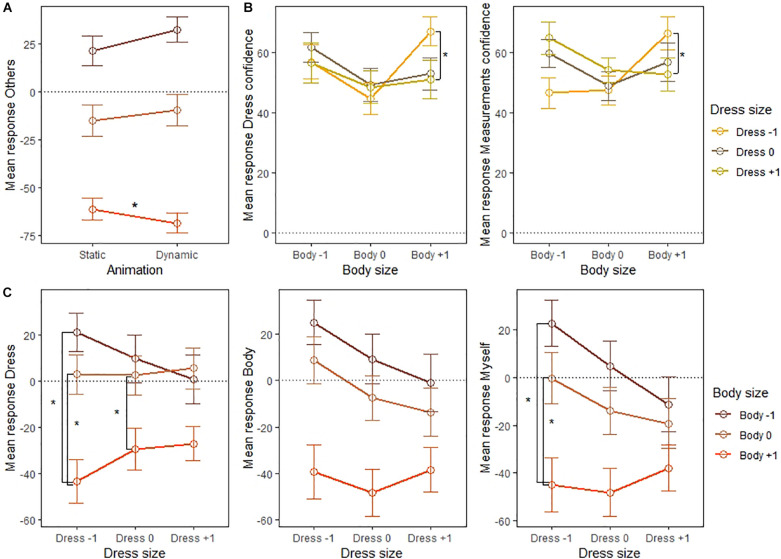
Interaction effects of **(A)** Animation and Body size for the Others item, **(B)** Body size and Dress size for the Dress confidence and Measurements confidence items, and **(C)** Body size and Dress size for the Dress, Body, and Myself items. Dress = *How likely do you think it is that this dress fits you?*, Dress confidence = *How certain are you?*, Measurements confidence = *How certain are you?* (In response to *How likely do you think it is that this avatar’s measurements correspond to your own?*), Body = *I feel as if the body of the avatar is my own body*, Myself = *The avatar reflects how I consider myself to be*, Others = *I consider the avatar to reflect how I want to present myself to others*. Body/Dress –1 = One body/dress size smaller than participants’ real body/dress size, Body/Dress 0 = Participants’ real body/dress size, Body/Dress +1 = One body/dress size bigger than participants’ real body/dress size.

An interaction between Animation and Body size was found for the “Others” item [*F*(2,12) = 4.26, *p* = 0.040, $\eta_{p}^{2}$ = 0.42, *BF*_10_ = 7.829e^+26^]. The difference between static and dynamic avatars was only significant for Body +1 (*t*(14) = 2.91, *p* = 0.033, *d* = 0.22; see Figure 3A), with dynamic avatars (*M* = −68.36, SD = 31.38) rated lower than static avatars (*M* = −61.20, SD = 32.52).

Furthermore, a two-way interaction between Body and Dress size was observed for the items “Dress” [*F*(4,10) = 3.87, *p* = 0.038, $\eta_{p}^{2}$ = 0.61, *BF*_10_ = 35373.918], “Dress confidence” [*F*(4,10) = 3.68, *p* = 0.043, $\eta_{p}^{2}$ = 0.60, *BF*_10_ = 0.074], “Measurements confidence” [*F*(4,10) = 4.87, *p* = 0.019, $\eta_{p}^{2}$ = 0.66, *BF*_10_ = 0.005], “Body” [*F*(4,10) = 4.25, *p* = 0.029, $\eta_{p}^{2}$ = 0.63, *BF*_10_ = 1128.508], and “Myself” [*F*(4,10) = 4.90, *p* = 0.019, $\eta_{p}^{2}$ = 0.66, *BF*_10_ = 506.754]. Figure 3B and Table 3 suggest that for the “Dress confidence” and “Measurements confidence” items, Dress −1 was rated significantly higher than Dress +1 for Body +1 only, suggesting that participants were more confident about their answers when presented with the biggest body size. Additionally, for the “Dress,” “Body,” and “Myself” items, the difference between Body +1 and the other body sizes was stronger for Dress −1 and Dress 0 compared to Dress +1 (see Figure 3C and Table 3; note that for the “Body” item, however, none of the comparisons survived correction).

Note that all significant interactions reached the threshold of moderate evidence to reject the null hypothesis, except for the items related to confidence of dress and measurements fit when looking at the interaction between Body and Dress size. No interactions between Animation and Dress size or three-way interactions were observed.

### Correlation Analyses With Body Esteem and Personality Questionnaires

**FIGURE 4:**
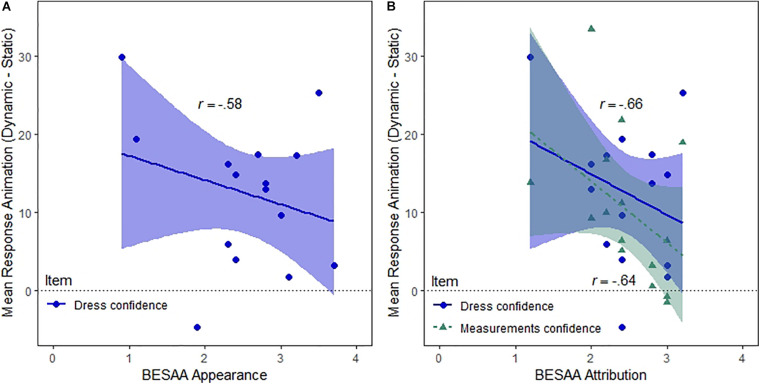
Correlations between the main effect of Animation and the **(A)** Appearance (general feelings about one’s appearance) and **(B)** Attribution (evaluations attributed to others about one’s body appearance) subscales of the Body Esteem Scale for Adolescents and Adults (BESAA). Higher scores on the BESAA reflect more positive attitudes. Dress = *How likely do you think it is that this dress fits you?*, Dress confidence = *How certain are you?*, Measurements confidence = *How certain are you?* (In response to *How likely do you think it is that this avatar’s measurements correspond to your own?*).

In order to reduce the number of tests, we restricted our correlation analyses with the body esteem and personality questionnaires to the main effect of Animation (Dynamic–Static) for all items, given that this was our main effect of interest. For the “Dress confidence” item, a significant negative relationship was observed for the Appearance (*r* = −0.58, *p* = 0.045) and Attribution (*r* = −0.66, *p* = 0.033) subscales of the BESAA, suggesting that the ratings difference between dynamic and static avatars for confidence about dress fit was bigger for participants with more negative feelings (see Figure 4A) and evaluations attributed to others concerning their own body appearance (and vice versa; see Figure 4B). A negative correlation was also found between the Attribution subscale of the BESAA and the “Measurements confidence” item (*r* = −0.64, *p* = 0.042), indicating that the same negative relationship existed when participants were asked to rate confidence about measurements correspondence (see Figure 4B). There were no other significant correlations for the effect of Animation (all *p*s > 0.06).

## Discussion

In the current study, we investigated own-body perception in a real-life practical setting by asking participants to match their own body with an externally perceived body that was a 3D-generated avatar based on participants’ real bodies, fitted with a computer-generated dress. This perceived body was (1) either static or dynamic, (2) either bigger, smaller, or the same size as participants’ own body size, and (3) fitted with a dress with a size either bigger, smaller, or the same as participants’ own dress size. Although we expected the addition of action cues (i.e., a walking avatar) to improve the ability to match own and an avatar’s body size (i.e., own body perception ratings), we only observed an effect of moving vs. non-moving avatars when participants had to indicate their confidence in their answer about whether the dress they had just seen would fit them (irrespective of the accuracy of their answer to the item on dress fit). Importantly, however, this observed difference between static and dynamic avatars was dependent on participants’ bodily self-esteem: participants with more negative feelings toward their own body felt more confident when confronted with dynamic avatars than participants with less negative feelings. Furthermore, when asked to rate how well the avatar reflected how participants wanted to represent themselves to others, we observed that dynamic avatars were rated lower than static avatars for the biggest-sized bodies only. For several self-report items, participants systematically rated smaller body/dress sizes higher than bigger body/dress sizes. When asked about confidence about dress and measurements fit, however, the higher ratings for smaller dress sizes were only present for the biggest body size. Finally, when participants had to rate dress fit, how strongly they felt that the avatar’s body was their own, and how the avatar represented how they considered themselves to be, the difference between the biggest body size and the other body sizes was strongest for the smallest dress sizes. We discuss these observed effects and potential limitations in more detail in the following sections.

### Effects of Animation

The role of the motor system in shaping and maintaining the bodily self and body ownership in particular has been well-documented by neuroimaging studies showing the emergence of premotor cortex activity lying at the root of our body schema (Graziano et al., 1994; Fogassi et al., 1996; Ehrsson et al., 2004, 2005; Convento et al., 2018), as well as body distortion illusions in healthy (Dummer et al., 2009; Vallar and Ronchi, 2009; Garbarini et al., 2013; Bolognini et al., 2014; Hara et al., 2015; della Gatta et al., 2016) and patient (Burin et al., 2015; Nava et al., 2017) populations. Thus, it seems that the sensory and motor system dynamically interact to develop our bodily self-awareness and self-consciousness (Nava et al., 2018). Interestingly, however, the influence of dynamic action cues on own-body perception when confronted with the task of matching own with an externally perceived body has thus far received little attention.

Our study, which compared dynamic and static avatars by adding walking animations (Varol et al., 2017), indicated that dynamic avatars were only rated higher than static avatars when participants had to rate the confidence in their answer concerning dress fit, suggesting that dynamic avatars increased participants’ certainty about dress fit irrespective of the accuracy of their answer to this item. Furthermore, this difference in ratings between walking and non-walking avatars was bigger for participants with low bodily self-esteem (in terms of confidence about both dress and measurement fit). The question that arises is what prompted participants with negative feelings toward their own body to feel more confident when confronted with dynamic avatars. It has been shown that people who tend to overestimate their own body measurements show disturbed fixation patterns when observing different bodies (Irvine et al., 2019), largely focusing on uninformative areas (Cornelissen et al., 2016). Our results indicate that people with low bodily self-esteem (commonly associated with over-estimation of own body size, see e.g., Ahadzadeh et al., 2018) might also focus their attention differently when dynamic action cues are added to observed avatars, possibly needing or caring more about the added value of these cues. Future research is warranted, however, to explore fixation patterns in own-body perception of dynamic bodies, and the influence of individual personality differences. Finally, when participants were asked to rate whether the avatar they were presented with reflected how they wanted to present themselves to others, dynamic avatars were rated lower than static avatars when they observed avatars with bigger-sized bodies. Thus, it seems that action cues lead to a lower preference of bigger-sized moving avatars when participants had to consider their bodies in a social context, possibly suggesting that movement dynamics cues are especially informative for bigger-sized bodies and consequently exacerbate the socio-cultural weight stigma (Fikkan and Rothblum, 2012; Spahlholz et al., 2016). While it has been shown that body image is partly a social construct (Davison, 2012), further research is needed to investigate the role of action cues in own-body perception, particularly when considering its social implications.

There are several reasons why our Animation manipulation might not have improved own-perceived body matching to the degree that we expected it to. First, it is possible that our static condition introduced implied motion. Previous research has shown that the observation of bodily actions employs visual (Grossman and Blake, 2002; Kable and Chatterjee, 2006) and motor areas (Rizzolatti and Craighero, 2004), even when motion is merely implied by static human postures (Urgesi et al., 2006, 2007; Candidi et al., 2008). Furthermore, it has been observed that body size and implied motion interact in influencing aesthetic appreciation of human bodies (Cazzato et al., 2012, Cazzato et al., 2016a), such that implied motion increases the aesthetic preference for thinner bodies (Cazzato et al., 2012). Thus, while the static condition in the current experiment did not offer the same action cues as the dynamic condition, the use of static human postures (representing dynamic movements) likely introduced implied motion of the observed bodies and dresses. Future research should address this important confound, and explore the contribution of dynamic cues when they are contrasted to a purely static condition. Second, an implicit measure of own-body recognition might have been more appropriate than our explicit self-report measure to access bodily representations that use motor/dynamic information. It has been shown that explicit and implicit recognition of our own body depend on different cortical mechanisms (Candini et al., 2016), and that only the former is based on motor information (Ferri et al., 2011). Thus, the explicit task in the current experiment might have only minimally relied on the dynamic cues provided by the Animation manipulation. Follow-up research using more implicit measures of own-body recognition is necessary to shed more light on this issue. Finally, we opted to manipulate movement dynamics by adding walking movements, rather than movements that people typically perform in front of a mirror (e.g., twisting and turning), because we believed they would be more informative and because they offer a viewpoint that we normally don’t (but probably would like to) have access to. However, the choice for these walking movements made the movement dynamics cues less compatible with real-life experiences, which may have affected our findings.

### Effects of Body Size

In line with previous research (Longo and Haggard, 2012; Hashimoto and Iriki, 2013; Kaplan et al., 2013; Linkenauger et al., 2017; Sadibolova et al., 2019; Maister et al., 2021), we observed that participants were unable to accurately identify their own body measurements. Furthermore, we replicated results from a previous study (De Coster et al., 2020), showing that participants – irrespective of their own body size – rate smaller-sized bodies higher (i.e., more attractive, more as a body that represents how you want to present yourself to others and that you would use for online shopping) than bigger-sized bodies, even when this own-perceived body matching takes place in a concrete context with practical implications. These findings, obtained using technology that was able to generate highly realistic avatar bodies (Loper et al., 2015), are in line with the body weight stigma that is especially pervasive in women (Fikkan and Rothblum, 2012; Spahlholz et al., 2016), and with research indicating that people tend to underestimate their body size (e.g., Monteath and McCabe, 1997; Tovée et al., 2003; Cazzato et al., 2015; Robinson and Kersbergen, 2017; Steinsbekk et al., 2017; Ralph-Nearman et al., 2019).

### Effects of Dress Size

Importantly, we fitted the different avatar bodies with different sizes of a highly realistic computer-generated dress (Narain et al., 2013; Pfaff et al., 2014) to further increase the experiment’s ecological validity and realism. Similar to the effect of body size, our results indicated that participants rated the smallest dress sizes higher than the bigger ones. This difference was only present for the biggest-sized bodies when participants had to rate confidence in their answers concerning dress and measurement fit, however, seemingly suggesting that the biggest body size made it easier for participants to discern the difference between the smallest and the biggest dress sizes. The same was true for the difference between the biggest and smallest body sizes, which was strongest for the smallest dress size for the “Dress” (*How likely do you think it is that this dress fits you?*), “Body” (*I feel as if the body of the avatar is my own*), and “Myself” (*The avatar reflects how I consider myself to be*) items. Together, these results indicate that own-body perception relies on a combination of an avatar’s body and clothing information when participants are presented with realistic avatars and garments. Thus, this suggests that garment fit and movement might provide important relevant cues for body size estimation. Importantly, however, the addition of these realistic, ecologically valid cues did not improve own-body perception in terms of the ability to match an externally perceived body with one’s own (contrary to Cornelissen et al., 2017), since participants remained unable to identify their own body and dress size accurately. It has to be noted, though, that both the main effect of dress size and its interaction with body size for the confidence items did not meet the threshold to reject the null hypothesis based on moderate evidence set during our Bayesian analysis, which suggests that these effects should be interpreted with caution and warrant further exploration.

### Limitations and Implications

The study has several important limitations. First, although BMI measures in the current sample were inside the “normal” or “healthy” range, we did not include any measures of pathological and/or negative body image, nor were participants excluded based on current or previous history of eating or body dysmorphic disorders. The influence of these disorders should be addressed in further research, since it has been shown that they greatly impact body size estimation (Tovée et al., 2003; Cornelissen et al., 2017). Second, the sample size in our study (15 participants) was relatively low. Due to several restrictions imposed by the COVID-19 pandemic at the time of the study, the available subject pool was limited (e.g., 360° videos of participants’ bodies had to be at our disposal). However, a Bayesian power analysis indicated that our sample was sufficiently large to answer our main research questions. Finally, it is important to note that we were unable to assess order effects related to the self-report items in the current study, given that the items were always presented in the same order (note that this does not apply to the order of the experimental conditions, which was randomized). Although this was done deliberately to make the task easier for participants, follow-up research should explore the possibility of order effects for the self-report items. Furthermore, the “Attractiveness” item (“*How attractive do you find the woman represented by this avatar?”*) could have been confusing to participants, given that they were informed that they would observe avatars based on their own body (but of different sizes). While this might have induced participants to self-evaluate their own perceived attractiveness, the observation that smaller-sized bodies were rated as more attractive than bigger-sized bodies seems to suggest that our manipulation was (at least partly) successful.

The influence of eating and/or body dysmorphic disorders on body size estimation is a topic of extensive research. Research suggests, for example, that body size overestimation is a defining feature of anorexia nervosa (Hennighausen et al., 1999; Gardner and Brown, 2014; Dakanalis et al., 2016; Gadsby, 2017; Malighetti et al., 2020; but see Cornelissen et al., 2013 who showed that body size overestimation in women with anorexia nervosa is not qualitatively different from the overestimation observed in women without anorexia nervosa), and that this overestimation is robust to manipulations that improve the accuracy of body size perception in healthy controls. While we expect that the addition of action cues might lead to stronger effects in clinical populations, in part suggested by the observation in the current study that participants with low bodily self-esteem showed an increased advantage of dynamic avatars, and based on previous studies that suggest that people with anorexia nervosa have a heightened sensitivity to visual bodily cues (Eshkevari et al., 2012; Keizer et al., 2014; Crucianelli et al., 2019; see Martinaud et al., 2017 for similar results in neurological patients), it is unclear which direction this influence would take (increased vs. decreased accuracy), especially given the fact that our Animation manipulation did not alter the accuracy of own-perceived body matching. However, as described above, future research should include screening for clinical disorders as well as more implicit measures in order to address the clinical implications of our findings better. Furthermore, the use of implicit tasks might also provide more information concerning the practical implications of the current research. Avatar design and development for online retail experiences, for example, depend on maximizing the congruency between the observed avatar and the self for better outcomes (e.g., greater purchase intentions, lower return rates; Kim and Forsythe, 2008). While dynamic cues did not increase accuracy of matching own with a perceived avatar’s body, research suggests that only implicit measures might be susceptible to such cues (Ferri et al., 2011).

## Conclusion

In sum, the current study aimed at contextualizing own-body perception in a real-life, practical situation by uniquely combining different technologies to create realistic, walking, dress-fitted avatars. None of these factors, however, seemed to improve own-perceived body matching, indicating that participants’ own body representations largely remain inaccurate (Hashimoto and Iriki, 2013; Linkenauger et al., 2017; Pitron and de Vignemont, 2017; Sadibolova et al., 2019; Maister et al., 2021) even in a realistic, concrete situation that has practical implications. These findings provide important insights for research exploring the development of online avatars (Kim and Forsythe, 2008) and research investigating own-body perception in clinical disorders such as anorexia nervosa and body dysmorphic disorders (e.g., Tovée et al., 2003; Cornelissen et al., 2017).

## Data Availability Statement

The original contributions presented in the study are included in the article/Supplementary Material, further inquiries can be directed to the corresponding authors.

## Ethics Statement

The studies involving human participants were reviewed and approved by the local ethics committee at the Universidad Carlos III de Madrid. The patients/participants provided their written informed consent to participate in this study. Written informed consent was obtained from the individual(s) for the publication of any potentially identifiable images or data included in this article.

## Author Contributions

All authors designed the research and reviewed the manuscript. LDC and PS-H developed the experimental stimuli. LDC implemented the experimental procedure, carried out the experiments, performed the analyses, and wrote the first draft of the manuscript.

## Conflict of Interest

The authors declare that the research was conducted in the absence of any commercial or financial relationships that could be construed as a potential conflict of interest.

## Publisher’s Note

All claims expressed in this article are solely those of the authors and do not necessarily represent those of their affiliated organizations, or those of the publisher, the editors and the reviewers. Any product that may be evaluated in this article, or claim that may be made by its manufacturer, is not guaranteed or endorsed by the publisher.

## Footnotes

1
https://www.clo3d.com/
